# Carriage of Extended Spectrum Beta Lactamase-Producing *Escherichia coli*: Prevalence and Factors Associated with Fecal Colonization of Dogs from a Pet Clinic in Lower Saxony, Germany

**DOI:** 10.3390/ani13040584

**Published:** 2023-02-07

**Authors:** Marco Werhahn Beining, Maria Hartmann, Antina Luebke-Becker, Sebastian Guenther, Katharina Schaufler, Katja Hille, Lothar Kreienbrock

**Affiliations:** 1 Department of Biometry, Epidemiology and Information Processing, WHO-Collaborating Centre for Research and Training in Veterinary Public Health, University of Veterinary Medicine, 27367 Hannover, Germany; 2 Centre for Infection Medicine, Institute of Microbiology and Epizootics, Freie Universität Berlin, 14163 Berlin, Germany; 3 Veterinary Centre for Resistance Research (TZR), Freie Universität Berlin, 14163 Berlin, Germany; 4 Pharmaceutical Biology, Institute of Pharmacy, University of Greifswald, 17489 Greifswald, Germany; 5 Pharmaceutical Microbiology, Institute of Pharmacy, University of Greifswald, 17489 Greifswald, Germany

**Keywords:** antibiotic resistance, risk factors, epidemiological study, raw food, nutrition, BARF, transmission, puppies, kennel

## Abstract

**Simple Summary:**

Among its role within the commensal bacterial flora, *Escherichia coli* (*E. coli*) is known as a cause of intestinal or extraintestinal diseases in pets and their owners. In order to reveal factors associated with the carriage of extended spectrum beta-lactamase-producing *Escherichia coli* in dogs, rectal swabs from 1000 dogs visiting a pet animal clinic in northern Germany within one year were tested. Additional data were sampled regarding, on the one hand, the dog’s health and husbandry conditions and, on the other hand, information about the owner´s medical history. Thus, we were able to define seven factors associated with extended spectrum beta-lactamase-producing *Escherichia coli* carriage. The high number of dogs tested and the exceptional data volume concerning the dog and owner itself, as well as those habits and interactions, underline the importance of our study to avoid the carriage and spread of pathogens, especially regarding the One Health aspect.

**Abstract:**

Extended spectrum beta-lactamase (ESBL)-producing *Escherichia coli* are an emerging problem in veterinary and human medicine. Our study concentrated on the estimation of the prevalence and factors associated with the carriage of ESBL-producing *E. coli* in dogs who visited a veterinary clinic in northern Germany in 2017. For this reason, 1000 patients (healthy and sick dogs) were tested, resulting in 1000 samples originating from rectal swabs. Additional data were collected using a self-reported questionnaire that was completed by the dog owner. Factors associated with ESBL carriage were considered for further modeling if *p* < 0.05 using a two-sided Fisher test. Using a backward elimination procedure, the variables for the final multivariable logistic regression model were identified. In total, 8.9% of the dogs tested were positive for carriage of ESBL-producing *E. coli*. Seven factors were associated with the colonization of dogs with ESBL-*E. coli* within the multivariable model, namely husbandry system (*p* = 0.0019, OR = 3.00; 95% CI: 1.50–6.00), contact with puppies (*p* = 0.0044, OR = 2.43; 95% CI: 1.32–4.46), feeding of raw meat (*p* = 0.011, OR = 2.28; 95% CI: 1.21–4.31), food residues (*p* = 0.0151, OR = 2.31; 95% CI: 1.18–4.53) and food supplements (*p* = 0.0487, OR = 0.426; 95% CI: 0.18–0.96), and antibiotic treatments of dogs (*p* = 0.0005, OR = 3.030; 95% CI: 1.62–5.68) or owners (*p* = 0.041, OR = 2.74; 95% CI: 1.04–7.19) prior to the study. These factors refer to the animals themselves as well as to the owners and their habits or medical treatments. Although the causality and direction of transmission from owners to their dogs cannot be proven, the factor of antibiotic treatment of the owner is clearly associated with the dog’s status.

## 1. Introduction

*Escherichia coli* (*E. coli*) belongs to the family of *Enterobacteriaceae* within the class *Gammaproteobacteria*. This gram-negative, rod-shaped bacterium is a main member of the aerobe commensal bacterial flora of dogs and other mammals. Its rapid growth capacity makes it appropriate for evolutionary studies with more than 50,000 generations [1,2,3]. *E. coli* has been identified as an indicator of antimicrobial selection pressure [4] and represents a potential reservoir of resistance determinants for other pathogenic or zoonotic bacteria [5,6]. Although *E. coli* is part of the commensal gastrointestinal flora of humans and dogs, it can cause diarrhea as well as extra-intestinal infections [7,8,9,10]. Close, direct contact between dogs and humans, such as petting, licking, physical injuries, and domestic conditions (e.g., feeding and furnishings), favors the possible zoonotic transmission of pathogens, particularly via the fecal-oral route [5,11].

Resistance to beta-lactam antibiotics in *E. coli* is frequently caused by hydrolyzing enzymes such as extended spectrum beta-lactamases (ESBL), which represent one of the most important resistance mechanisms in gram-negative bacteria [12]. ESBL is usually associated with *E. coli*. In recent reports, other *Enterobacteriaceae* such as *Klebsiella* spp., *Citrobacter*, *Serratia*, *Proteus*, *Salmonella*, and *Enterobacter* having ESBL were shown [13] in dogs with diarrhea as well as in healthy dogs. ESBL-producing strains from *Enterobacteriaceae* were found, which originated from humans and companion animals, leading to the assumption of a possible transmission between humans and companion animals [14].

CTX-M-type ESBLs were first detected in a laboratory dog in Japan in 1988 [15]. In recent decades, ESBL-producing *E. coli* have spread all over the world in clinical settings as well as in livestock, wildlife, and the environment [16]. In 2000, Teshager et al. reported the isolation of ESBL-producing *E. coli* from a dog with recurrent urinary tract infections, which was one of the first cases observed in companion animals [17].

This occurrence of antimicrobial-resistant bacteria within the pet population causes two general problems. On the one hand, the increase in antimicrobial resistance (AMR) may lead to limitations regarding therapeutic options for veterinarians, which could possibly end in a treatment crisis. Marques et al. [18] report a magnitude of prevalence that may indicate that these restrictions may result in therapeutic restrictions on antibiotic usage in pet care.

The transmission of MDR bacteria from livestock to humans and the subsequent spread of these pathogens to family members, as described by Nienhoff et al., 2009 emphasizes the high degree of contagiousness of these bacteria between animals and humans [19]. On the other hand, considering the “One Health” concept, strategies to prevent outbreaks of ESBL-producing *E. coli* strains and other MDR pathogens in veterinary clinics must be improved to protect future patients as well as veterinary personnel and animal owners [20]. In particular, persons belonging to higher-risk groups, such as immunosuppressed patients, children, pregnant women, aged people, and patients with chronic diseases [11], are more susceptible to opportunistic infections that are associated with ESBL-producing *E. coli*, such as urinary tract infections [21,22], bacteremia [23,24] or even rare diseases, such as endocarditis [25,26]. Consequently, harboring intestinal ESBL-producing *E. coli* can lead to serious infections in animals and humans with fundamental therapeutic complications and serious health restrictions for the animals themselves and their owners [18,27].

Data on the prevalence of ESBL-producing *E. coli* in companion animals is very ambiguous. Generally, studies have reported a prevalence that ranges from 0.5% to approximately 50% with different study designs (e.g., enrollment of ill or pretreated patients), laboratory methods, or questionnaire information (for details see discussion) Systematic studies on the situation in Germany are lacking. We aimed to obtain basic data on the occurrence of ESBL-producing *E. coli* in a dog population typical of small animal hospitals (a mixture of healthy and diseased dogs) and to investigate factors associated with the colonization of dogs with ESBL-producing *E. coli*. Therefore, dog- and owner-related factors were assessed.

## 2. Materials and Methods

### 2.1. Study Design and Inclusion of Participants

This cross-sectional study investigated the prevalence of ESBL-producing *E. coli* in the dog population of a clinic in northern Germany. This pet clinic serves mostly as a referral center, with a smaller part serving as a general practitioner with 50 employees and treating about 6500 patients annually. Between October 2016 and December 2017, every owner who visited the clinic received a flyer giving information about the study and a personal briefing on the aims of the study and the study design. Dog owners agreed to participate by signing an informed consent form, including a privacy statement. This is in line with German ethical guidelines. No other inclusion or exclusion criteria were applied. Rectal swab samples were taken from 1000 dogs regardless of whether they were healthy or not.

### 2.2. Questionnaire

Every dog owner was asked to complete a questionnaire containing three parts. The first and second parts included information about the dog, such as breed, age, gender, country of origin in case of imported dogs, the reason for the visit, husbandry conditions and contact with other animals or people working in healthcare settings, prior diseases and treatment, diet, and general customs. Husbandry conditions have been divided into keeping within the household (“indoor dogs”) and keeping only outdoors (“outdoor dogs”). Regarding diet, the questionnaire differentiated, inter alia, dry and wet food, raw food, and food residues. Supplements were defined as, e.g., vitamins and minerals. The method of keeping only outdoors includes husbandry in a shelter or garden as well as in any auxiliary building, such as a stable. The third part contained information about the owner regarding general chronic diseases and prior antibiotic treatments, contact with diarrhea patients, nutritional habits, occupation, gender, and living area. Following the interview, the data were immediately checked by the veterinarian responsible for the study for missing information and general queries from the owner. The complete questionnaire (version in the German language) is available at https://www.tiho-hannover.de/kliniken-institute/institute/bioepi/publikationen/zusatzmaterial-publikationen/ (accessed on 3 October 2022).

The questionnaire was audited beforehand, and comprehensibility for the dog owner was assessed. The duration of the interview was less than 10 min.

All data were entered into a Microsoft Access 2003 study database.

### 2.3. Sample Collection and Laboratory Processing

Rectal swabs (Amies-Medium with charcoal, Mastaswab, Mast Diagnostica Reinfeld, Germany) were taken exclusively for this study by the responsible veterinarian prior to the examination. All swabs were kept at 8 °C until further processing. Bacterial isolation and identification were performed by the Institute of Microbiology and Epizootics, Centre for Infection Medicine, Freie Universität Berlin.

All swabs were streaked onto self-made CHROMagar Orientation plates prepared from commercial chrome agar powder (Mast Diagnostica, Reinfeld, Germany) with added cefotaxime (2 µg/mL, Merck, Darmstadt, Germany). Cefotaxime was added after autoclaving and cooling the medium close to solidification, followed by mixing to ensure a correct concentration. Freshly prepared plates were incubated at 37 °C for 24 h. According to the manufacturer’s protocols, colonies showing an *E. coli*-like phenotype were subcultured. Purification of the colonies was performed using the identically prepared ChromAgar Orientation plates.

Single colonies were obtained via two subsequent subcultures for species identification by using MALDI-TOF MS (MALDI Microflex ^®^ LT and Biotyper database ^®^; Bruker Daltonics, Bremen, Germany). Phenotypical resistance examination was performed using the Vitek2 Compact System (card: VITEK^®^AST-GN98 [bioMérieux, Craponne, France], reference strain: *E. coli ATCC 52922*).

Confirmatory tests were performed applying the standard CLSI guideline method [CLSI guideline VET01 2018]. For the testing of beta-lactam antibiotics, the common standard reference and quality control strain *E. coli ATCC 35218* [28] was used.

Molecular confirmation of ESBL production was performed for all isolates, using specific ESBL and plasmid ampC primers by polymerase chain reaction [29].

### 2.4. Statistical Analysis

Based on the microbiology results, specimens were classified as “positive” for verified ESBL carriers and “negative” for cases of no verification. These outcomes were analyzed statistically for their associations with the questionnaire responses calculating odds ratios applying the two-sided Fisher test. If variables with fewer than five observations per category occurred, the categories were aggregated, or the variable was excluded from further modeling due to sparse data. Afterward, a multivariable logistic regression model without interaction terms was considered with all variables from the univariable analyses with a *p*-value < 0.05, and a backward elimination process was applied for the final model. To avoid multi-collinearity within the model, Cramer’s V was calculated for all pairwise associations of the factors included. However, no V > 0.4 was observed. Therefore, all factors were used for modeling.

A resulting *p*-value of <0.05 was considered statistically significant with no multiple adjustments due to the exploratory nature of the study. All data were analyzed using the software SAS, version 9.4 TS Level 1M5 (Statistical Analysis System^®^, SAS Institute Inc., Cary, NC, USA).

## 3. Results

### 3.1. Study Population

Dog owners who presented their dogs to the veterinary clinic during the recruitment period (October 2016–December 2017) were asked for permission to have their dogs participate in this study. Approximately 40% of all dogs treated were enrolled to take part in the study. Those dogs that were presented for prevention were more likely to participate. A total of 1000 dogs were enrolled. Nearly half of the tested dogs were male (n = 461). The median dog age was 59.9 months (range 1–198 months). Most dogs were purebred (63.5%), and the Labrador Retriever was the most common breed (n = 65). A total of 89 different breeds were represented. Almost 75% (n = 774) of the dogs included in the study were raised in Germany. Approximately half (48.7%) of the tested dogs belonged to a household with more than one dog.

Approximately half of the patients (n = 566) presented due to any disease, whereas 434 dogs visited the clinic for preventive reasons, such as deworming or vaccination. Ophthalmic (n = 111) and orthopedic diseases (n = 96) were most frequent, which were followed by internal diseases (n = 87), follow-up consultation (n = 71), and surgery (e.g., soft tissue, joints, and fractures, n = 45). Other reasons for the visits were dental (n = 19), dermal (n = 19), and gastroenterological diseases (n = 33), as well as patients who presented to the departments of gynecology (n = 33), oncology (n = 36) and the least, were emergency cases (n = 26, e.g., trauma or intoxication).

The majority of dogs lived in rural areas (n = 693), and slightly less than 10% lived in large cities (n = 58). In total, 12.8% of the owners worked in the agricultural sector.

The percentage of dogs that were referred by other veterinarians was 15.7%, and 29.9% suffered from an acute disease. Antibiotic treatment during the previous three months prior to sampling was administered to 176 of the 1000 studied dogs.

Dog owners were mainly female (72.2%). Regarding medical issues, 320 owners had previous contact with (human or animal) patients with diarrhea in the last 12 months, while 109 of these were with human patients and 238 were with animals. Less than 5% of the owners had suffered from diarrhea four weeks prior to the study, and 43 of all owners had received antibiotic treatment two months prior to taking their dogs part in the study (for further details, see Table 1).

Considering the age distribution, the prevalence was higher in younger dogs. Dogs aged under 12 months (n = 185) showed a prevalence of 11.9% (n = 22), and dogs aged between 12 and 24 months (n = 134) had a prevalence of 7.4% (n = 10). During the middle age between 24 and 48 months (n = 174), the prevalence drops to 5.2% (n = 9) and increases again in advanced age over 48 months (n = 508) to 9.6%.

### 3.2. Status of ESBL-Carriage and Basic Questionnaire Variables

All 1000 owners completed the questionnaires and gave full access to dogs’ samples. From these, ESBL-producing *E. coli* (ESBL-*E. coli*) was isolated from 89 rectal swabs. The results of the univariable analysis of the questionnaire variables with the outcome of the sample results are summarized in Table 1 (see supplements for detailed results for all variables).

More than half of the variables related to the husbandry conditions of the tested dogs appeared to be statistically significant (*p* < 0.05) in the univariable model. Among those, there are some outstanding *p*-values of factors such as “keeping the dog outdoors’ (*p* > 0.0001), ‘contact with puppies’ (*p* < 0.0001), ‘staying in a shelter’ (*p* = 0.0004), and ‘multidog household’ (*p* = 0.0007).

In particular, those variables that concerned feeding practices resulted in small *p*-values. According to that, `feeding raw meat (*p* = 0.0029), food residues (*p* = 0.0044), feeding treats (*p* = 0.0002), and supplements (*p* = 0.02) were statistically significant factors.

Considering medical histories, a *p*-value of <0.0001 was observed for contact between a tested dog and any diarrhea patient (e.g., human, dog, or other animals).

Regardless of whether the dog or owner had received antibiotic treatment within the last three months prior to sampling, both were associated with ESBL-*E. coli* carriage of the dog (dog treated: *p* = 0.0003; owner treated: *p* = 0.0185).

The final multivariable model revealed seven factors that were associated with colonization with ESBL-producing *E. coli* (see Table 2). These factors can be assigned to the areas of how dogs were kept, feeding habits (especially raw meat, leftover food, and food supplements), and antimicrobial treatment of dogs and owners.

Feeding habits such as the feeding of raw meat, food residues, and food supplements play an important role as risk factors for the carriage of ESBL *E. coli.* In addition, treatment with antibiotics of the dog or the owner also presents relevant risk factors in the multivariable model. Similarly, husbandry conditions, like keeping dogs outdoors and contact with puppies, were two relevant factors for ESBL-*E. coli* isolation from dog samples (see Table 2)

## 4. Discussion

Overall, an occurrence rate of 8.9% of ESBL-*E. coli*-positive samples was observed within this study population. The occurrences of ESBL-*E. coli* has been associated with factors related to husbandry, feeding habits, and the medical histories of dogs as well as their owners.

Since 1000 dogs were systematically recruited for this study, to the best of the author´s knowledge, this is one of the largest studies that identifies the factors associated with ESBL-producing *E. coli* carriage in an ordinary dog population visiting a veterinary clinic (including preventive examinations).

However, due to the voluntary participation of the dog owners, the results of our study are possibly prone to selection bias. Therefore, the study population was compared with the overall clinic population. Dogs that were presented for prevention were more likely to participate (43.3%). Normally, only 25% of patients visit the clinic for preventive care and show no clinical signs of disease (calculation based on data from the clinic patient management software). This may lead to a bias in the resistance prevalence. The average age in the study population was slightly younger (59 months vs. 69 months in usual patients). Regarding the age distribution, the prevalence drops from younger dogs to middle age before it increases in dogs older than 48 months. Thus, our study might have underestimated the prevalence.

However, nearly 45% of the presented dogs were male, which was a proportion similar to the usual clinical population. Furthermore, the percentage of cross-breed dogs was very similar to the entire clinical population (34.7% of the clinical population versus 36.5% of the tested dog population). Overall, it may be stated that the sample is a representative sub-group of the clinic population, which itself is identified as a typical set of dog patients in the northern parts of Germany.

Due to the diagnostic procedures used, the risk of bias based on the microbiological methods used in contrast to other study results was low. The framework of this study includes only the testing of dogs without considering the possible ESBL *E. coli* carriage of the owner. Thus, the transmission from owner to dog or vice versa cannot be proven.

The questionnaire´s basic concept has been successfully used in different studies [30,31] and was therefore elaborated based on these experiences. Therefore, the occurrence of any information bias due to the questionnaire information was avoided.

To deal with multi-collinearity, Cramer’s V was calculated for all pairs of factors to identify pairwise associations. Within this procedure, no extended V > 0.4 was observed, which led to the inclusion of all factors in the multivariable model.

Various factors that were associated with ESBL-producing *E. coli* colonization in the univariable model could not be confirmed by the multivariable model, which generally emphasizes the need for confounding adjustment by means of multivariable statistical models. For example, there was a noticeable *p* = 0.0012 when dogs originated from abroad in the univariable model. However, this factor was not statistically significant in the multivariable model. This is in line with Rzewuska et al., as well as with other studies from neighboring countries, which confirm an increase in the pan-European presence of ESBL-producing *E. coli*, even if there were deficiencies in the comparisons among uni- and multivariable statistical analyses in these investigations [32,33].

It is a significant finding that the odds ratios that remained in the final multivariable model were stable when compared to the univariable calculations. Along with the statistical procedures, these results indicate that the included factors suggest only a little or no confounding, i.e., the factors do not influence each other. In addition, the OR of all factors remained over 2, which is a general twofold effect or greater for all factors identified.

Though the prevalence of 8.9% of ESBL-producing *E. coli* in dogs is generally in agreement with other studies (see Table 3), these studies used different inclusion or exclusion criteria, and the kind of study population varied considerably. 

In our study, the healthy subpopulation showed a higher prevalence than the diseased ones. This is not in line with other studies which reported higher prevalence within a diseased dog population [9,53].

Concerning the geographical aspect, Albrechtova et al. [54] and Wedley et al. [34] examined a dog population in a rural and semirural area with different outstanding prevalence of 75% and 0.5%. Focusing on these two extremes, the region of living could be quite relevant regarding the carriage of ESBl-producing *E. coli*.

Comparing dog populations visiting veterinary practices, Wedley et al. [34] found a quite low prevalence of 1.9%, which is lower than our result. Hospitalized dogs were excluded, and samples were taken from many practices, which does not allow a direct comparison with our study. Higher than in our study is the prevalence in a study completed in a veterinary clinic in Turkey [30], Tunisia [51], and China [53]. Here the prevalence were considerably higher than in our study (16.8%, 19.5%, and 41.3%). One reason could be different treatment regimens and resulting resistance prevalence.

Even German study results vary greatly. Schmiedel et al. (2014) reported a high prevalence of 52% when testing infected dogs (e.g., isolates taken from different sources, for example, routine screening for ESBL-producing *E. coli*, blood cultures, or urine samples) [47], while Ewers et al. (2010) reported that ESBL-producing *E. coli* had been isolated from 10.7% of clinical samples that were collected from dogs with urinary tract infections (UTIs), wound infections and diarrhea [41]. In contrast to these results, Schmidt et al. reported a prevalence of 1.4% when testing clinically healthy dogs [55]. It needs to be considered that these studies handled clinical samples while we studied the colonization of dogs.

In addition, isolation methods and the detection of the ESBL-producing phenotypes/genotypes have been described insufficiently in many studies, which limits direct comparisons of the study results [56].

Considering the heterogeneity of the study in Table 3, it gets apparent that the majority of publications of infected and possibly treated dogs show higher percentages of ESBL-*E. coli* carriers than studies that include healthy, non-treated dogs. This emphasizes the urgent need for a standardized protocol that enables a comparison of risk factors, possibly within a systematic review.

Seven factors were statistically significantly associated with the carriage of ESBL-producing *E. coli* in the multivariable model: keeping methods, contact of dogs with puppies, feeding of raw meat and food residues, antibiotic treatment of dog and owner, and, surprisingly, the protective role of giving feeding supplements.

### 4.1. Husbandry Conditions

The OR of 2.99 from our study and publications that have analyzed this factor [45,52,53] shows the importance of this factor in the carriage of ESBL-producing *E. coli*. In summary, 24 (19.8%) of the 121 dogs that were kept only outdoors were ESBL-*E. coli* carriers. In contrast, only 7.44% of dogs primarily kept indoors (n = 65) tested positive. Studies that have compared keeping conditions are quite rare. Usually, studies of multidog households involve breeders, who generally keep their dogs in kennels (“outdoor dogs”). De Graef et al. found more antimicrobial-resistant *E. coli* in kennel dogs than in privately owned dogs [57]. Similar results were reported by Belas et al. [44], who compared dogs from shelters/breeders and privately owned dogs. The findings of Harada, which indicate that the spread of multiresistant *E. coli* extends beyond different litters and affects the entire kennel [58], emphasize the transmission risk in multidog households. In particular, dogs originating from the same kennel have close contact in their outdoor pens and, thus, a common environment, which can contribute to infections by multiresistant bacteria [59]. Nevertheless, it has also been shown that some dogs remained ESBL-*E. coli* negative even though several of their littermates in the same household were confirmed to be ESBL-*E. coli* positive [30].

### 4.2. Contact with Puppies

Since the factors ‘keeping predominantly outdoors’ and ‘contact with puppies’ are associated with a higher risk of carrying ESBL-*E. coli*, it may be assumed as a surrogate for breeding dogs and a higher prevalence in these. In addition to the findings of higher incidences of antimicrobial-resistant *E. coli* in breeding dogs [57], the frequent misuse of antibiotics in breeding kennels is associated with the occurrence of multidrug-resistant bacteria [59]. Dogs represent a potential reservoir for pathogenic *E. coli* [60,61,62]. Thus, the transfer of bacteria or resistance determinants from mother to puppy can occur through the milk and vaginal flora as well as through contact with feces. In their study, Münnich and Lübke-Becker determined that vertical transfer was the most common route for *E. coli* infections in puppies. Similarly, ESBL-producing *E. coli* can be transferred among dogs and their owners through close contact [41,42,63].

The easy vertical transfer between bitches and puppies and, consequently, even subclinical infections of the puppies could explain why 14.6% of the 321 dogs that had contact with puppies in the last 12 months prior to the study were positive for ESBL-producing *E. coli* [63].

Dogs having contact with puppies are believed to have a higher risk for infection with ESBL *E. coli*, which is reflected in our results. It is probable that dogs kept by breeders have, on the one hand, regular contact with puppies and, on the other hand, are kept in groups. In general, keeping animals in groups or herds could increase the risk of spreading antimicrobial-resistant bacteria within such populations [58,64,65].

### 4.3. Feeding Customs

Currently, it has become popular in many countries to feed dogs with ‘BARF’ (‘biologically appropriate raw food’ or ‘bones and raw food’) [66,67] that originates from game and livestock. Higher prevalence values of *E. coli*-positive samples have been found among commercial raw pet foods when compared with conventionally processed foods [68,69]. In addition, individual diets have been supplemented with freeze-dried products, such as pig ears or tracheas. These products are usually raw and may pose a considerable risk of contamination by pathogens [69,70,71,72]. Studies have shown a connection between feeding raw food and the carriage of ESBL-producing *E. coli*. Thus, Leonard et al. reported that feeding raw meat or adding anything raw to the diet is associated with a higher risk of colonization with ESBL-producing *E. coli* [73], which is supported by the results of this study.

Van Bree et al. were able to detect ESBL-producing *E. coli* in 80% of analyzed commercial raw meat-based diets [74], and Hellgren et al. found *Enterobacteriaceae* in 100% of their samples [75]. Storing and preparation of raw foodstuffs may also pose health risks to persons who come into contact with the feed, which may pose a risk of transmission [67], especially for patients who belong to high-risk groups [66,75].

Another possible source of bacterial transmission in addition to pet food is sharing of food that is intended for human consumption. Naziri et al. (2016) reported that feeding raw meat as well as sharing human food with their own dogs poses a high risk of infection [76]. Our results confirm the findings of previous studies related to the potential transmission of ESBL-producing *E. coli* within the food chain in pet food and through feeding or handling of raw meat.

### 4.4. Antibiotic Treatment

The selection of ESBL-producing strains is promoted by antibiotic use, which has significant implications for current therapeutic options and the epidemiologic resistance situation [77]. Our findings are supported by other studies, which showed a considerably higher risk of carrying ESBL-producing *E. coli* after antimicrobial treatments [6,44].

Schmidt et al. described the prevalence of ESBL-producing *E. coli* immediately after treatment in dog feces as well as at one and three months after treatment. Although this study found an association between antimicrobial treatments and 3GCR (third-generation cephalosporin-resistant)/AmpC, no evidence showed an association between antimicrobial treatments and ESBL-producing *E. coli*. Nevertheless, the study indicated a higher likelihood of carrying AMR *E. coli* up to one month after the end of antimicrobial therapy [78].

Similar results have also been described in several studies regarding ESBL-producing *E. coli* in humans after antimicrobial treatments [79,80], especially in the case of inappropriate use of antibiotics [81]. Even up to three months after antimicrobial treatments, a higher risk for carriage of ESBL-producing *Enterobacteriaceae* was observed [37].

Thus, the association between antibiotic treatments and the carriage of ESBL-producing *E. coli* has been described and emphasized in several publications, which is supported by our study.

### 4.5. Feeding Supplements

Our results suggest that the provision of dietary supplements (e.g., vitamins and minerals) has a protective effect on dogs. Unexpectedly, dogs receiving feed supplements had a lower likelihood of being colonized with ESBL-producing *E. coli*. However, the *p*-value of 0.0487 is quite low.

Studies regarding the protective effects of food supplements, especially in small animal medicine, are quite rare. Furthermore, the association between the habits of owners who feed supplements and their influence on the dissemination of ESBL-producing *E. coli* is unclear.

The reasons for this result could only be estimated. Possibly, owners who are used to feeding supplements care more for their dog’s health. The interaction between dogs and supplement-feeding owners needs further investigation to better understand our findings.

## 5. Conclusions

Our analyses showed in our study population an association between different factors, such as feeding raw meat and antibiotic treatments, with ESBL-producing *E. coli* colonization in dogs. Moreover, we identified risk factors such as “contact with humans with diarrhea” and “antibiotic intake of the owner”, which suggest that transmission from human to dog and vice versa seems likely. With regard to a One Health approach, doctors of medicine as well as veterinarians should be aware of this possible transmission and its effects.

## Figures and Tables

**Table 1:** Basic questionnaire variables * and association with ESBL carriage in the univariable model. CI = 95% confidence interval from Wald; *p* = *p*-value from linear logistic regression.

Factor (Time Period)	Category	Total	ESBL-pos		ESBL-neg		OR	CI	*p*
		n	n	%	n	%			
Demographic characteristics of the dogs									
Gender of the dog	Male	461	36	7.8	425	92.2	1		
	Female	539	53	9.8	486	90.2	1.29	0.83–2.01	0.2635
Origin	Germany	774	56	7.2	718	92.8	1		
	Abroad	225	33	14.7	192	85.3	2.2	1.39–3.49	0.0007
Reason of visit	Prevention	434	52	12.0	382	88.0	1.95	1.25–3.03	0.0031
	Sick	566	37	5.5	529	93.5	1		
Husbandry conditions									
Multidog household	No	513	30	5.8	483	94.2	1		
	Yes	487	59	12.1	428	87.9	2.22	1.40–3.51	0.0007
More animals: small mammal	No	863	65	7.5	798	92.5	1		
	Yes	137	24	17.5	113	82.5	2.61	1.57–4.33	0.0002
More animals: cat	No	710	50	7.0	660	93.0	1		
	Yes	290	39	13.5	251	86.5	2.05	1.32–3.2	0.0015
More animals: horse	No	830	61	7.3	769	92.7	1		
	Yes	170	28	16.5	142	83.5	2.47	1.54–4.03	0.0002
More animals: pig	No	918	75	8.2	843	91.8	1		
	Yes	82	14	17.1	68	82.9	2.31	1.24–4.3	0.0082
Keeping form	Indoor	874	65	7.4	809	92.6	1		
	Outdoor	121	24	19.8	97	80.2	3.08	1.84–5.15	<0.0001
Dog contact with:	Small animals	739	65	8.8	674	91.2	1.36	0.73–2.5	0.33
	Other animals	58	11	19.0	47	81	3.3	1.39–7.82	0.0069
	No contact	196	13	6.6	183	93.4	1		
Dog contact with cat	No	720	53	7.4	667	92.6	1		
	Yes	280	36	12.9	244	87.1	1.86	1.83–2.91	0.0068
Contact with puppy (12 months)	No	658	41	6.2	617	93.8	1		
	Yes	321	47	4.6	274	85.4	2.46	1.57–3.82	<0.0001
Lick owners face (12 months)	Never/rare	680	49	7.2	631	92.8	1		
	Regularly	97	7	7.2	90	92.8	1.00	0.44–2.28	0.997
	Daily	180	32	17.8	148	82.2	2.79	1.72–4.50	<0.0001
Stay in Shelter (12 months)	No	848	67	7.9	781	92.1	1		
	Yes	122	22	18.0	100	82.0	2.57	1.52–4.33	0.0004
Feeding: raw meat	No	728	54	7.4	674	92.6	1		
	Yes	246	34	13.8	212	86.2	2.00	1.27–3.16	0.0029
Feeding: food residues	No		66	7.9	766	92.1	1		
	Yes	142	22	15.5	120	84.5	2.13	1.27–3.58	0.0044
Feeding: treat	No	305	43	14.1	262	85.9	2.30	1.48–3.58	0.0002
	Yes	675	45	6.7	630	93.3	1		
Feeding: supplements	No	754	78	10.3	676	89.7	1		
	Yes	201	10	5.0	191	95.0	0.45	0.23–0.89	0.0223
Medical history dog									
Acute disease	No	662	70	10.6	592	89.4	1		
	Yes	299	19	6.4	280	93.6	0.57	0.34–0.97	0.0387
Antibiotic treatment (3 months)	No	797	60	7.5	737	92.5	1		
	Yes	176	29	16.5	147	83.5	2.42	1.50–3.91	0.0003
Contact with any diarrhea patient (12 months)	No	413	27	6.5	386	93.5	1		
	Yes	231	39	16.9	192	83.1	2.90	1.73–4.89	<0.0001
	Unknown	256	23	6.5	333	94.5	0.99	0.57–1.76	0.9656
Contact with human with diarrhea (12 months)	No	931	77	8.3	854	91.7	1		
	Yes	69	12	17.4	57	83.6	2.34	1.20–4.54	0.0124
Contact with animals with diarrhea (12 months)	No	823	60	7.3	763	92.7	1		
	Yes	177	29	16.4	148	83.6	2.49	1.55–4.02	0.0002
Demographic characteristics and medical history owner									
Living environment	Small city	229	11	4.8	218	95.2	1		
	Rural area	693	74	10.7	619	89.3	2.37	1.23–4.55	0.0095
	Large city	58	4	6.9	54	93.1	1.47	0.45–4.79	0.5246
Gender	Male	233	12	5.1	221	94.9	1		
	Female	723	74	10.2	649	89.8	2.1	1.12–3.94	0.0207
Profession	Other	631	41	6.5	590	93.5	1		
	No Profession	200	23	11.5	177	88.5	1.87	1.09–3.20	0.0225
	Agricultural sector	128	24	18.8	104	81.2	3.32	1.93–5.73	<0.0001
	Unknown	41	1	2.4	40	97.6	0.36	0.05–2.68	0.3187
Contact with diarrhea patient (12 months)	No	257	15	5.8	242	94.2	1		
	Yes	320	46	14.4	274	85.6	2.71	1.48–4.97	0.0013
	Unknown	423	28	6.6	395	93.4	1.14	0.6–2.19	0.6844
Contact with human with diarrhea (12 months)	No	891	74	8.3	817	9.69	1		
	Yes	109	15	13.8	94	86.24	1.76	0.97–3.19	0.0619
Contact with animal with diarrhea (12 months)	No	762	55	7.2	707	92.8	1		
	Yes	238	34	14.29	204	85.71	2.14	1.36–3.38	0.001
Suffered from diarrhea (4 weeks)	No	902	73	8.1	829	91.9	1		
	Yes	49	8	16.3	41	83.7	2.22	1.00–4.90	0.0496
	Unknown	49	8	16.3	41	83.7	2.22	1.00–4.90	0.0496
Antibiotic treatment (2 months)	No	912	73	8	839	92	1		
	Yes	43	8	18.6	35	81.4	2.63	1.77–5.88	0.0185

* Full data can be accessed at https://www.tiho-hannover.de/kliniken-institute/institute/bioepi/publikationen/zusatzmaterial-publikationen/ (accessed on 3 October 2022).

**Table 2:** Questionnaire variables and association with ESBL-*E. coli* carriage in the final multivariable model.

Factor	Category	Final Multivariable Model		
		OR	CI	*p*
Husbandry conditions				
Keeping form	Household	0.0001		
	Other	3.00	1.50–6.00	0.0019
Contact with puppy last 12 months	No	0.0001		
	Yes	2.43	1.32–4.46	0.0044
Feeding: raw meat	No	0.0001		
	Yes	2.28	1.21–4.31	0.0110
Feeding: Food residues	No	0.0001		
	Yes	2.31	1.18–4.53	0.0151
Feeding: supplements	No	0.0001		
	yes	0.426	0.18–0.96	0.0487
Medical history dog				
Antibiotic treatment last 3 months	No	0.0001		
	Yes	3.03	1.62–5.68	0.0005
Medical history owner				
Antibiotic treatment last 2 months	No	0.0001		
	Yes	2.74	1.04–7.19	0.041

**Table 3:** Brief summary of recent studies on ESBL-producing *E. coli* in small animals.

Country	%	Reference	Year	Study Details
European Studies				
Cheshire, UK	0.5	[34]	2011	n = 183, dogs in a semi-rural community
UK	1.9	[35]	2017	n = 580, dogs visiting a veterinary practice
Denmark	1.9	[36]	2015	n = 209, fecal samples from public garden
Switzerland	2.9	[37]	2013	n = 174, nursing homes and vet practice, Enterobacteriaceae
Poland	3.4	[33]	2015	n = 119, veterinary faculty, diseased dogs
Switzerland	3.4	[38]	2013	n = 59 dogs, urinary samples
Italy	4	[39]	2005	n = 298, dog/cat/rat, healthy, diseased, dead
Portugal	7.8	[40]	2008	n = 39, healthy pets
Germany	10,7	[41]	2010	n = 84 diseased dogs, mainly German samples
Germany	12	[42]	2015	n = 100, fecal sample from veterinary faculty and neighborhood
France	12.8	[43]	2014	n = 368, healthy dogs, veterinary clinic
Portugal	13.2	[44]	2014	n = 151 vet. clinic, not infected dogs, Lisbon
Turkey	16.8	[30]	2017	n = 428, healthy dogs, veterinary clinic
Spain	17.8	[45]	2018	n = 140 dogs, samples from laboratories, 25 positive samples, no further patient data
Netherland	50	[46]	2013	n = 40, 50% diarrhea patients, ESBL/AmpC
Germany	52.2	[47]	2014	n = 67 dogs, infected patients
Non-European Studies				
USA	3	[48]	2011	n = 944, dogs and cats, samples from veterinary practitioners
Mexico	6	[49]	2015	n = 53, healthy dogs in a public area
Algeria	14.7	[50]	2016	n = 102 healthy dogs, veterinary practice
Tunisia	19.5	[51]	2013	n = 41, veterinary clinic, healthy dogs
USA	26.7	[9]	2010	n = 150, dogs and cats, samples from UTI
Korea	33.3	[52]	2012	n = 63 hospitalized dogs
China	41.3	[53]	2010	n = 240 dog/cat, veterinary clinics/pet shop
Angola	75	[54]	2014	n = 17, stray dogs, no medical history known

## Data Availability

Full data can be accessed at https://www.tiho-hannover.de/kliniken-institute/institute/bioepi/publikationen/zusatzmaterial-publikationen/.
